# rTMS and TENS Relieve Neuropathic Pain in CCI Model Rats by Modulating Central Nervous System TRPV1 and Neuroinflammation

**DOI:** 10.1155/mi/8500317

**Published:** 2024-11-21

**Authors:** Zhangyu Xu, Quanzhen Zhong, Fei Xing, Yuanliang Zhu, Yue Hu, Maomao Huang, Mouwang Zhou, Jianxiong Wang

**Affiliations:** ^1^Department of Rehabilitation Medicine, The Affiliated Hospital of Southwest Medical University, Luzhou, Sichuan, China; ^2^Department of Rehabilitation Medicine, Southwest Medical University, Luzhou, Sichuan, China; ^3^Rehabilitation Medicine and Engineering Key Laboratory of Luzhou, Luzhou Science and Technology Bureau, Luzhou, Sichuan, China; ^4^Department of Rehabilitation Medicine, Zigong First People's Hospital, Zigong, Sichuan Province, China; ^5^Department of Rehabilitation Medicine, Peking University Third Hospital, Beijing, China; ^6^Department of Clinical Medicine, Southwest Medical University, Luzhou, Sichuan, China

**Keywords:** inflammatory factors, neuroinflammation, neuropathic pain, rTMS, TENS, TRPV1

## Abstract

**Background:** Repetitive transcranial magnetic stimulation (rTMS) of the prefrontal cortex (PFC) and transcutaneous electrical nerve stimulation (TENS) have both been demonstrated as effective at alleviating neuropathic pain (NP). However, the comparative efficacy of these two neuromodulation techniques and the specific neural mechanisms underlying their effects remain unclear.

**Objective:** This study aims to compare the efficacy of rTMS in the PFC and TENS in mitigating peripheral NP and to investigate the impact of rTMS on neuroinflammation.

**Methods:** Eighteen adult male Sprague–Dawley rats were randomly divided into three groups: NP (chronic constriction injury [CCI] group, *n* = 6), rTMS (*n* = 6), and TENS (*n* = 6). rTMS was applied to the PFC, while TENS was applied to the right hind limb of the rats 1 week postoperatively. This treatment regimen was administered once daily, 5 days a week, for 4 consecutive weeks. The paw withdrawal mechanical threshold (PWMT) was measured to assess the pain-alleviating effects of rTMS and TENS. We further conducted enzyme-linked immunosorbent assays (ELISAs) to measure the levels of interleukin (IL)-1β, IL-6, and tumor necrosis factor alpha (TNF-α) in the PFC and L4–L6 spinal cord to evaluate their impact on neuroinflammation. Additionally, we examined transient receptor potential vanilloid type 1 (TRPV1) expression in the PFC and the L4‒L6 spinal cord using western blotting and real-time quantitative reverse transcriptase polymerase chain reaction (qRT-PCR) to explore the potential mechanisms involved. Hematoxylin and eosin (H&E) staining of the sciatic nerve was further performed to observe pathological changes.

**Results:** Compared to the CCI group, both the rTMS and TENS groups exhibited a significant increase in PWMT, with the rTMS group demonstrating a notably greater PWMT than the TENS group. Furthermore, rTMS treatment triggered a significant decrease in IL-1β, IL-6, and TNF-α levels in the PFC and spinal cord, while TENS only decreased IL-1β expression in these regions. In both treatment groups, TRPV1 expression was significantly lower in the spinal cord, while H&E staining indicated improved pathological manifestations in the sciatic nerve.

**Conclusion:** Both rTMS and TENS effectively ameliorated CCI-induced NP, with rTMS of the PFC showing superior performance. Both treatments reduced TRPV1 expression and suppressed neuroinflammation in the spinal cord, indicating that this may be one of the mechanisms through which they exert their therapeutic effects.

## 1. Introduction

According to the International Association for the Study of Pain, neuropathic pain (NP) is defined as pain caused by a lesion or disease of the somatosensory nervous system [1]. NP affects ~7%–8% of individuals and is characterized by pain hypersensitivity and hyperalgesia, which can significantly impair physical, emotional, psychological, and social functions [1–3]. Various treatment options are currently available, including pharmacotherapy with calcium channel modulators and antidepressants, neuromodulation therapy, and minimally invasive interventional therapies. Furthermore, exercise therapy and some pain self-management strategies have been considered to help relieve pain [4, 5]. However, achieving satisfactory control of NP remains a complex clinical challenge.

Repetitive transcranial magnetic stimulation (rTMS), a representative treatment modality for central nervous system modulation, and transcutaneous electrical nerve stimulation (TENS), a strategy designed to modulate peripheral nerves, have both shown promise in NP management [6–8]. However, the comparative efficacy of these two interventions (particularly rTMS in the left prefrontal cortex [PFC]) remains uncertain, and further research is needed to elucidate their specific neural mechanisms.

Neuroinflammation is closely associated with NP development. As such, reversing or reducing inflammation is an effective strategy for controlling NP [9, 10]. By targeting the cerebral cortex, rTMS modulates neuroinflammation and partially reverses mechanical nociception in rats with peripheral nerve injury [11]. In our previous study, we found that inflammatory factors were significantly increased in the PFC of rats with chronic constriction injury (CCI) and that rTMS of the PFC region improved the NP and simultaneously downregulated the expression of inflammatory factors in the PFC region [12]. TENS, a peripheral nerve stimulation technique, can reduce inflammatory cytokines and relieve pain in rats with peripheral nerve injury [13]. As such, controlling neuroinflammatory responses in the central nervous system (CNS) through central or peripheral regulation may represent a common mechanism of action for rTMS and TENS.

Transient receptor potential vanilloid type 1 (TRPV1) is a ligand-gated, nonselective Ca^2+^ channel that plays a central role in the nociceptive hypersensitivity associated with NP, making it a key feature of neuroinflammation [14–16]. In neuroinflammation-related diseases, inflammatory cytokines enhance the activity and expression of TRPV1, which regulates the release of inflammatory cytokines [14, 17, 18]. Consequently, TRPV1 is a potential target for the treatment of neurological disorders. Numerous studies have further shown that the modulation of TRPV1 by pharmacological targeting can alleviate NP and ameliorate neuroinflammation [19–22]. Given that both rTMS and TENS modulate peripheral NP function and ameliorate neuroinflammation, coupled with our pre-experimental findings indicating their modulatory effects on TRPV1, we hypothesized that rTMS and TENS can affect NP function resulting from peripheral nerve injury through the regulation of TRPV1.

As such, this study aimed to compare the effects of rTMS and TENS in treating NP and to explore whether the mechanisms of these interventions are related to central nervous system neuroinflammation through TRPV1 regulation. Further, we believe that this study provides valuable insights into the mechanisms of various neuromodulatory approaches in NP management.

## 2. Experimental Procedure

### 2.1. Animals

Eighteen adult male Sprague–Dawley rats weighing 220‒240 g and aged 8‒10 weeks were used as models in this study. Rats were housed in three polypropylene cages with sawdust covering the floor. The rats were housed in a familiar environment with access to water and rodent chow ad libitum. We used only male rats in this experiment as hormone levels in female rats have been shown to potentially alter the modulation of nociceptive responses. All procedures were approved by the Ethics Committee of Southwestern Medical University (Approval No. 20211115-003), in accordance with the guidelines of the International Association for the Study of Pain.

### 2.2. Experimental Design

CCI was induced in rats, which were subsequently randomly divided into three groups: NP group (CCI group, *n* = 6), rTMS group (*n* = 6), and TENS group (*n* = 6). The establishment of NP was verified using the Von Frey test 7 days after CCI surgery. Rats in the rTMS and TENS groups received rTMS in the PFC and TENS in the operated lower limb daily for 4 weeks, respectively, while rats in the CCI group were routinely maintained without any treatment. Mechanical nociception was assessed in all rats every 2 days using Von Frey filaments. After 4 weeks of intervention, all rats were sacrificed and dissected to obtain the brain, spinal cord, and sciatic nerve for further biochemical assays.

### 2.3. NP Model

NP was induced in the right sciatic nerve [23]. First, rats were anesthetized intraperitoneally with 1.0% sodium pentobarbital (40 mg/kg). An incision was made in the skin 3–4 mm below the right femur, and the muscle was bluntly separated to expose the sciatic nerve trunk. Double ligation was subsequently performed at the proximal end of the sciatic nerve bifurcation with four turns spaced 1 mm apart. Finally, the muscle and skin were sutured layer-by-layer.

### 2.4. rTMS Intervention

While research has shown that both low- and high-frequency rTMS can alleviate NP injury in model rats [11, 24], the optimal rTMS parameters for alleviating NP remain unclear. In our previous study, we compared the efficacy of 4 weeks of rTMS in the PFC at 1 versus 10 Hz, finding that rTMS at 10 Hz was superior to 1 Hz. Therefore, we used 10 Hz rTMS in the present study. One week after surgery, the rTMS group started receiving rTMS stimulation (6 cm diameter, 3 T peak intensity of the pulsed magnetic field) while anesthetized and immobilized on a rat stereotactic device. As in our previous studies, we set the coordinates to area postrema (AP) = *A* + 11.6 and medial lemniscus (ML) = + 4.75 [12]. Before treatment, resting motor thresholds were measured to standardize the treatment parameters. The rats were placed on a treatment table, and their heads were tightly immobilized during treatment. rTMS treatment was then performed using a circular coil in a direction tangential to the scalp at a location in the PFC. The other parameters used were the same as those in our previous study, as follows: stimulation frequency, 10 Hz; stimulation time, 20 s; interval time, 10 s; and stimulation intensity, 100% of the resting potential threshold (RMT), 1000 pulses. Treatment was administered once a day, 5 days a week for 4 weeks.

### 2.5. TENS Intervention

The optimal parameters for TENS for the treatment of NP are also unclear based on a meta-analysis by Huang et al. [25]. Therefore, choose the most commonly utilized treatment parameters, 100 Hz, and an intensity of submotor threshold, and each treatment lasted 10 min. TENS was also conducted once a day, 5 days a week for 4 weeks, similar to rTMS. Self-adhesive surface electrodes of 45 mm (length) and 5 mm (width) were applied to the chemically removed skin of rats around the knee, as well as on the thigh of the right hind limb. All rats were attached to the electrodes in the same position under the same conditions.

### 2.6. NP/CCI Group

The living environment and experimental procedures for rats in the CCI group were the same as those in the two treatment groups, the only difference being that no corresponding intervention was performed.

### 2.7. Paw Withdrawal Mechanical Threshold (PWMT) Test

**Figure 1:**
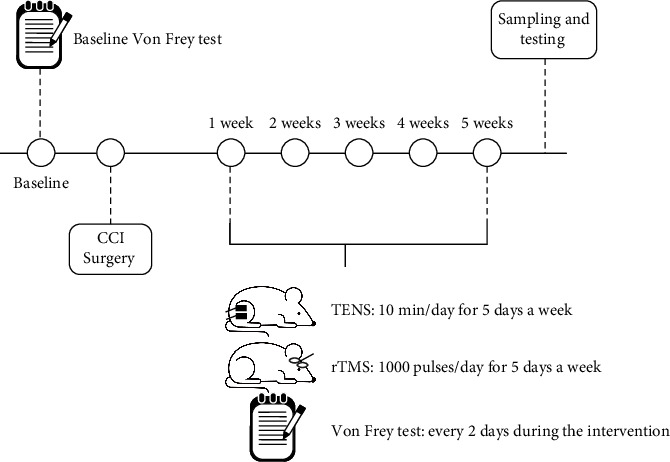
Experimental flow chart. CCI, chronic constriction injury; rTMS, repetitive transcranial magnetic stimulation; TENS, transcutaneous electrical nerve stimulation.

The PWMT was measured using Von Frey filaments. The test time points are shown in Figure 1, that is, 1 day before surgery (baseline), the seventh day after surgery, and every 2 days during the intervention. First, the rats were placed in a plastic cage with a wire mesh bottom and acclimated to the environment until quiet. The Von Frey filaments were subsequently placed perpendicular to the surface of the central hind paw of the rat foot and slightly curved at the bottom of the foot. The stimulations were spaced a few seconds apart and held for approximately 5 s. The response was considered positive if the rat performed rapid paw retraction or licked its foot. Instead, stimulation should be repeated. Withdrawal reflexes exceeding three stimulation grams were considered the response threshold for mechanical stimulation.

### 2.8. Biochemical Assays

Five weeks after surgery, the PFC and L4‒L6 spinal cord were collected from rats by decapitation and analyzed for enzyme-linked immunosorbent assay (ELISA) analysis of tumor necrosis factor alpha (TNF-α), interleukin (IL)-1β, and IL-6 and western blot analysis of TRPV.

### 2.9. ELISA for TNF-α, IL-1β, and IL-6

Rat ELISA kits for TNF-α (ZCIBIO Technology Co., Ltd., Shanghai, China), IL-1β (ZCIBIO Technology Co., Ltd., Shanghai, China), and IL-6 (ZCIBIO Technology Co., Ltd., Shanghai, China) were used to detect levels of TNF-α, IL-1β, and IL-6, respectively, in accordance with the manufacturer's instructions. The targeting antibody was encapsulated in a 96-well microtiter plate to create a solid-phase carrier, and standards or samples were added to the microtiter wells where the target was attached to the antibody bound to the solid-phase carrier. Next, the horseradish peroxidase-labeled antibody was added, the unbound antibody was removed, and the tetramethylbenzidine (TMB) substrate was added again for color development. The color shade is positively correlated with the target in the sample. The absorbance was measured at 450 nm using an enzyme marker to calculate the concentrations of TNF-α, IL-1β, and IL-6 in the samples.

### 2.10. Western Blotting for TRPV1

As in the previous methods, the PFC of the brain and spinal cord samples were removed and placed in 2 mL grinding tubes with 3 mm steel beads and radioimmunoprecipitation assay (RIPA) lysis solution (Beyotime Biotechnology, Shanghai, China) at a mass ratio of sample:lysis solution = 1:10, after which they were dissociated in a high-speed low-temperature tissue grinder (temperature of −20°C, 4 times, 60 s each time). The samples were removed, placed in a refrigerator at 4°C for 30 min for lysis, after which they were removed and put into a centrifuge (4°C, 12,000 rpm, 10 min). After centrifugation, the supernatant was removed, and the protein concentration was determined using a bicinchoninic acid (BCA) protein quantification kit (Beyotime Biotechnology, Shanghai, China). Following electrophoresis, the proteins in the gel were transferred to a polyvinylidene fluoride (PVDF) membrane (Sigma‒Aldrich, Shanghai, China) and then blocked with 5% bovine serum albumin (BSA) at room temperature for 1 h. Afterwards, the PVDF membrane was incubated overnight at 4°C in a dilution containing anti-TRPV1 (1:2000, ABclonal, Wuhan, China) and anti-β-actin (1:100,000, ABclonal, Wuhan, China) antibodies. The PVDF membrane was then washed three times with tris-buffered saline containing Tween-20 for 5 min each. The PVDF membrane was then incubated with a secondary antibody (diluted 1:5000) at room temperature for 2–3 h. The PVDF membrane was covered with a luminescent liquid (liquid A : liquid B; 1:1) and exposed to an enhanced chemiluminescence luminometer to obtain images. The test strips were exposed and scanned using the Tanon GIS chassis control software V20.0 (Tanon Technology Co., Ltd., Shanghai, China), and the results are expressed as the relative expression of the target protein, calculated as follows: Relative expression of target protein = integral optical density (IOD) value of target protein/IOD value of the internal control.

### 2.11. Real-Time Quantitative Reverse Transcriptase Polymerase Chain Reaction (qRT-PCR) for TRVP1

RNA was extracted from the samples using TRIzol reagent (Bomei, China) and subjected to reverse transcription using the PrimeScript RT reagent Kit (Takara Bio, China). Subsequently, qRT-PCR was conducted using TB Green Premix Ex Taq II (Tli RNase H Plus) (Takara Bio, China), using specific primers. The qRT-PCR protocol included initial denaturation at 95°C for 30 s, followed by 45 cycles of denaturation at 95°C for 5 s, annealing at 55°C for 30 s, and extension at 72°C for 30 s. The data were normalized to those of β-actin, and the relative mRNA expression levels were determined using the 2^−*Δ*CT^ method. The primer sequences are listed in Table 1.

### 2.12. Hematoxylin and Eosin (H&E) Staining

Following the intervention, the sciatic nerve was removed and fixed in fixative solution. Dehydration, embedding, sectioning, and H&E staining were subsequently performed. Images of the sections were acquired, the sections to be observed were selected, and images at ×100 magnification and ×400 magnification were collected to observe the pathology.

### 2.13. Statistical Analysis

SPSS 22.0 software statistical version 22.0 was used for analysis. Rats that died prior to analysis were excluded. Eighteen rats (CCI group = 6, TENS group = 6, rTMS group = 6) were included in the statistical analysis. All values are expressed as the mean ± standard deviation (mean ± SD). Western blotting and ELISA results were analyzed using one-way analysis of variance (ANOVA). PWMT results were analyzed using repeated-measures analysis of variance, and differences were considered statistically significant at *p* < 0.05.

## 3. Results

### 3.1. rTMS and TENS Improved the PWMT in CCI Rats

**Figure 2:**
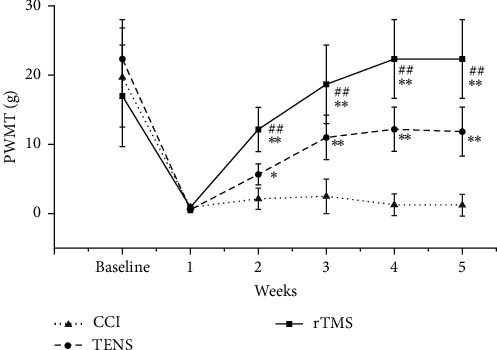
Changes in the paw withdrawal mechanical threshold (PWMT) of rats in each group (*⁣*^*∗*^*p* < 0.05 and *⁣*^*∗∗*^*p* < 0.01 vs. the chronic constriction injury [CCI] group; ^##^*p*  < 0.01 vs. the transcutaneous electrical nerve stimulation [TENS] group). rTMS, repetitive transcranial magnetic stimulation.

Figure 2 shows the PWMT changes in all rats during the experiment. One week after CCI surgery, the PWMT significantly decreased in all three groups, with no significant differences observed among the groups. Over the course of the 4-week treatment period, PWMT gradually increased in the rTMS group, reaching a statistically significant difference compared with values in the NP and TENS groups (*p*  < 0.01). In the TENS group, the PWMT was greater in the second week than in the CCI group (*p*  < 0.05), with a highly significant difference noted in weeks 3–5 compared with the CCI group (*p*  < 0.01).

### 3.2. The Effect of rTMS and TENS on IL-1β, IL-6, and TNF-α in the PFC and Spinal Cord

**Figure 3:**
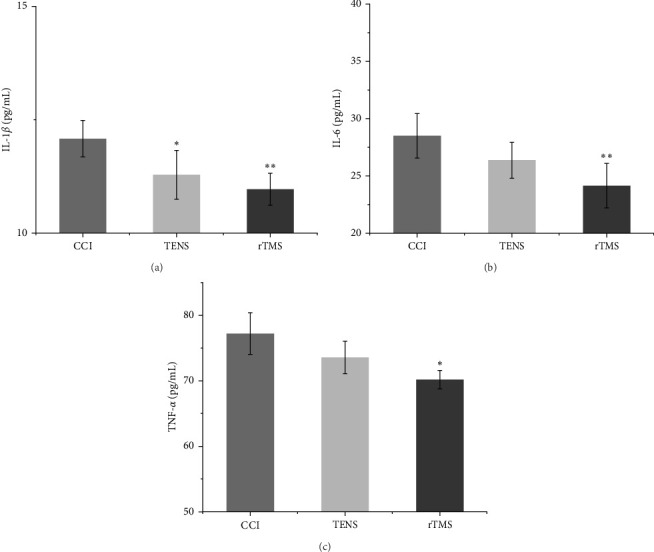
Changes in inflammatory factors in the spinal cord among the three groups: (A) shows the interleukin (IL)-1β expression level, (B) shows the IL-6 expression level, and (C) shows the tumor necrosis factor alpha (TNF-α) expression level (*⁣*^*∗*^*p* < 0.05 and *⁣*^*∗∗*^*p* < 0.05 vs. chronic constriction injury [CCI] group). rTMS, repetitive transcranial magnetic stimulation; TENS, transcutaneous electrical nerve stimulation.

**Figure 4:**
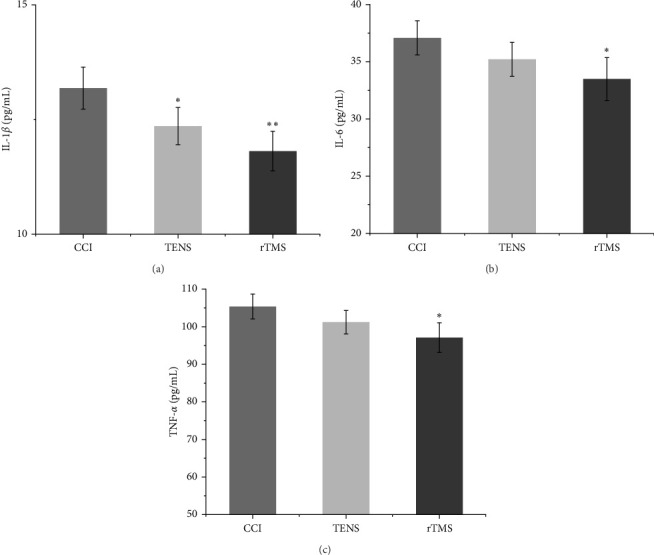
Changes in the levels of inflammatory factors in the prefrontal cortex (PFC) among the three groups: (A) shows the interleukin (IL)-1β expression level, (B) shows the IL-6 expression level, and (C) shows the tumor necrosis factor alpha (TNF-α) expression level (*⁣*^*∗*^*p* < 0.05 and *⁣*^*∗∗*^*p* < 0.05 vs. chronic constriction injury [CCI] group). rTMS, repetitive transcranial magnetic stimulation; TENS, transcutaneous electrical nerve stimulation.

ANOVA revealed significant differences in inflammatory factor levels among the three groups (Figures 3 and 4). After 4 weeks of rTMS intervention, the levels of IL-1β, IL-6, and TNF-α in the PFC and spinal cord were decreased compared with those in the CCI group. In the TENS group, all three inflammatory factors in the PFC and spinal cord decreased, although only IL-1β showed a significant reduction compared to the CCI group (*p*  < 0.05). In the comparison between the two interventions, rTMS exhibited a more substantial reduction in IL-1β, IL-6, and TNF-α than TENS.

### 3.3. Effect of rTMS and TENS on TRPV1 in the PFC and Spinal Cord

**Figure 5:**
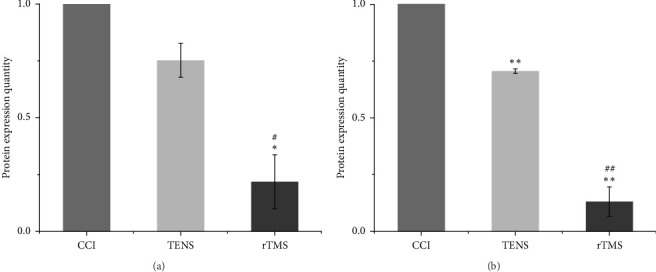
Western blotting results of the relative transient receptor potential vanilloid type 1 (TRPV1) protein expression: (A) shows the relative expression of TRPV1 in the prefrontal cortex (PFC), while (B) shows the relative expression in the spinal cord (this shows the integral optical density [IOD] rectified histogram, using the chronic constriction injury [CCI] group rectified IOD as the standard for comparison; *⁣*^*∗*^*p* < 0.05 and *⁣*^*∗∗*^*p* < 0.01 vs. the CCI group; ^#^*p* < 0.05 and ^##^*p* < 0.01 vs. the transcutaneous electrical nerve stimulation [TENS] group). rTMS, repetitive transcranial magnetic stimulation.

**Figure 6:**
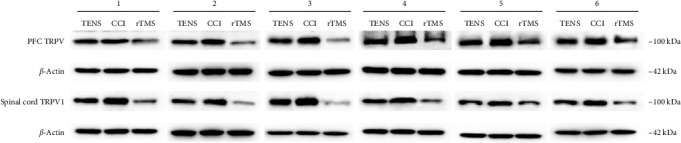
Transient receptor potential vanilloid type 1 (TRPV1) protein bands (numbers 1–6 indicate different replicates of the western blotting test). CCI, chronic constriction injury; PFC, prefrontal cortex; rTMS, repetitive transcranial magnetic stimulation; TENS, transcutaneous electrical nerve stimulation.

Statistical analysis indicated significant differences in TRPV1 expression among the three groups (Figure 5).  Figure 6 shows the TRPV1 protein bands on western blotting. Compared to the CCI group, the rTMS group exhibited reduced TRPV1 expression not only in the PFC (*p*  < 0.05), but also in the spinal cord (*p*  < 0.01) (Figure 5A,B), whereas TENS alone reduced TRPV1 expression in the spinal cord (*p*  < 0.01) (Figure 5B). Furthermore, rTMS led to a more marked reduction in TRPV1 levels in the PFC and spinal cord than TENS.

### 3.4. rTMS and TENS Ameliorated TRPV1 Gene Expression in the PFC and Spinal Cord

**Figure 7:**
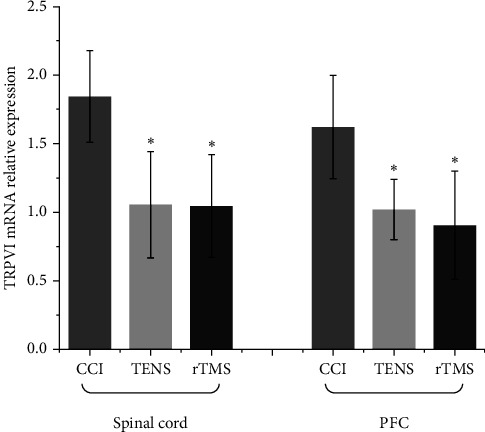
Real-time quantitative reverse transcriptase polymerase chain reaction (qRT-PCR) to detect transient receptor potential vanilloid type 1 (TRPV1) mRNA relative expression (*⁣*^*∗*^*p*  < 0.05 vs. the chronic constriction injury [CCI] group). PFC, prefrontal cortex; rTMS, repetitive transcranial magnetic stimulation; TENS, transcutaneous electrical nerve stimulation.

The mRNA expression of the TRPV1 gene in the PFC and spinal cord of CCI-induced NP model rats is shown in Figure 7. In the PFC and spinal cord, both rTMS and TEN downregulated TRPV1 gene expression (*p*  < 0.05, compared with the CCI group), but there was no difference in gene expression between the rTMS and TENS groups.

### 3.5. rTMS and TENS Improved Sciatic Nerve Pathology Injury

**Figure 8:**
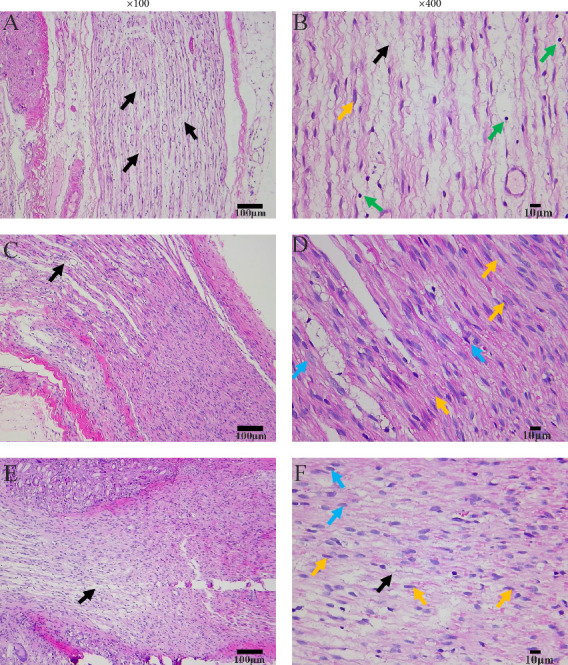
Analysis of the sciatic nerve by hematoxylin and eosin (H&E) staining light microscopy in the fifth week. (A), (B) Staining of the chronic constriction injury (CCI) group at ×100 magnification and ×400 magnification. (C), (D) Staining of the transcutaneous electrical nerve stimulation (TENS) group at ×100 magnification and ×400 magnification. (E), (F) Staining of the repetitive transcranial magnetic stimulation (rTMS) group at ×100 magnification and ×400 magnification. In all images, black arrows represent axonal breaks, green arrows represent lymphocytes, yellow arrows represent Schwann cells, and blue arrows represent fibroblasts.

rTMS and TENS improve sciatic nerve pathology in rats with CCI. The sciatic nerve tissue structure in the CCI group was intact and clear, with varying degrees of axonal breaks accompanied by a decrease in Schwann cells and an increase in inflammatory cells (Figure 8A,B). In the TENS group, the sciatic nerve fibers showed slight degenerative necrosis. There were a small number of axonal breaks, as well as proliferation of Schwann cells and fibrous tissue (Figure 8C,D). In the rTMS group, there was a minor number of axonal breaks in the sciatic nerve tissue and a large number of Schwann cells and fibrous tissue hyperplasia (Figure 8E,F). Compared with those in the CCI group, the sciatic nerve tissues in the rTMS and TENS groups showed a reduction in axon dissection, as well as different degrees of Schwann cell and fibrous tissue proliferation, indicating that the degree of sciatic nerve histopathological damage was reduced in both groups and that the difference in the degree of pathology between the two groups was not significant.

## 4. Discussion

This study represents the first attempt to compare the effects of rTMS and TENS on NP in CCI rats, focusing on their effects on TRPV1 expression and neuroinflammatory factors within the brain and spinal cord. These findings indicate that rTMS significantly outperformed TENS in alleviating mechanical pain in rats with NP. Furthermore, we observed that rTMS substantially downregulated TRPV1 expression in the PFC and spinal cord of CCI rats compared to rats treated with TENS. Concurrently, rTMS led to a reduction in the expression of inflammatory factors, namely, IL-1β, IL-6, and TNF-α. These results underscore the superior analgesic efficacy of rTMS in CCI rats compared to that of TENS. Notably, as the duration of the rTMS treatment increased, we observed a progressive improvement in the mechanical pain threshold of the rats, which was only partially reversed by TENS.

rTMS, a well-established neuromodulation technology, has been widely applied for the treatment of various functional disorders caused by conditions such as stroke, spinal cord injury, and related ailments such as hemiplegia, aphasia, spasticity, and cognitive dysfunction [26–28]. Furthermore, rTMS has demonstrated promising results in the management of both central and peripheral NP [11, 24]. Most notably, TMS targeting the M1 area, which is recognized as a frontline treatment for NP in this region, is considered a first-line treatment [29]. Although relatively limited research has focused on the PFC, some studies have investigated the effects and potential mechanisms of rTMS on the PFC for NP management, yielding encouraging findings. A systematic review by Zang et al. [30] reported that the left dorsolateral frontal cortex (DLPFC) is a potential target for rTMS in the treatment of NP. Furthermore, the application of rTMS to the premotor cortex/dorsolateral PFC in patients with spinal cord injury can relieve NP [31]. Consistent with these findings, our study supports the notion that rTMS targeting the PFC can effectively alleviate NP.

Conversely, TENS also effectively alleviates NP by exerting its influence on peripheral nerves. TENS is recommended as the first-line treatment for peripheral NP [6]. High-frequency ipsilateral TENS relieves mechanical nociception [13, 32, 33]. However, no previous studies have directly compared the differential effects of PFC-targeted rTMS and TENS on alleviating peripheral NP. Our findings clearly demonstrate that rTMS applied to the PFC surpassed TENS in improving PWMT and downregulating the expression of TRPV1 and inflammatory factors in CCI rats. Nevertheless, it is important to acknowledge that research regarding the application of rTMS to the PFC for NP treatment remains somewhat limited and that the optimal treatment parameters warrant further investigation.

In the present study, we specifically targeted the PFC for rTMS intervention. The PFC plays a pivotal role in pain processing because it maintains intricate connections with various regions of the neocortex, hippocampus, periaqueductal gray matter, thalamus, amygdala, and basal nucleus of the brain [34–36]. During acute and chronic pain, changes in neuroinflammation, glial cells, neurotransmitters, and gene expression occur in the PFC, leading to variations in its activity, connectivity, and structure [35]. Stimulation of the PFC has been shown to effectively suppress withdrawal reflex and aversive responses to pain in animal models [37–41]. Additionally, research has demonstrated that neuromodulatory strategies applied to the PFC effectively controlled chronic inflammatory pain in rats [42]. High-frequency rTMS targeting the cerebral cortex has shown promise in alleviating NP [43–45]. Our previous study revealed that rats with NP exhibited depression-like behavior and that the PFC was also closely related to mood. In addition, we found that application of 10 Hz rTMS of the left dorsal anterior insula (part of the PFC) was shown to be effective in alleviating mechanical hyperalgesia and reversing despair-like behavior in sciatic CCI rats. Based on these findings, we chose the PFC as the site for rTMS stimulation, which yielded satisfactory results.

Furthermore, our investigation revealed that both rTMS and TENS can mitigate NP by inhibiting the central inflammatory response within the nervous system. Notably, both interventions reduced the levels of classic inflammatory factors in the PFC and spinal cord. NP arises from the interplay between inflammatory cytokines and chemokines, mediated by neurons, glial cells, and a multitude of immune cells within the nervous system. Inflammatory factors play a pivotal role in peripheral NP by influencing local inflammatory responses and sensitivity of primary afferent neurons to pain [46, 47]. When peripheral NP occurs, the inflammatory signals generated can be transmitted to the PFC, hippocampus, and other supraspinal areas, resulting in elevation of central neuroinflammatory cytokines, such as IL-1β, IL-6, and TNF-α [11, 48]. Of these, IL-1β and TNF-α are critical mediators of neuroinflammation [49]. Following nerve injury, there is an increase in the expression of IL-1β and TNF-α, which act upon neurons and other inflammatory mediators, indirectly fostering the development of the NP [9]. IL-6 also appears to be associated with NP, with animal studies indicating a local increase in the mRNA and protein levels of IL-6 following peripheral nerve injury [50, 51]. Research has further underscored the pivotal role of inflammatory cytokines in the central nervous system in NP [51–53]. Previous findings have shown that high-frequency rTMS relieves NP by reducing the levels of inflammatory factors, such as IL-1β, IL-6, and TNF-α, in the brain [12]. Moreover, the application of rTMS in CCI rats effectively modulate neuroinflammation [11]. In addition, early TENS reduced the levels of inflammatory cytokines in the spinal cord of rats with NP [54]. Furthermore, our study revealed that rTMS effectively reduced the levels of all three inflammatory factors within the brain and spinal cord, whereas TENS only reduced the levels of IL-1β. This disparity may explain why rTMS provides superior pain relief.

Our study also yielded one significant discovery regarding the effect of rTMS and TENS on the reduction of TRPV1 within the PFC and spinal cord. TRPV1 receptors are widely distributed throughout the central and peripheral nervous systems, and their expression has been observed in all sensory ganglia, including the dorsal root ganglia (DRG), trigeminal, and vagal ganglia. Additionally, they are expressed in small sensory C and Aδ fibers, where they modulate responses to noxious stimuli and integrate various pain signals [55–57]. Following nerve injury, TRPV1 excitability increases, subsequently activating neuroinflammatory signaling pathways [14, 16]. TRPV1 receptors, often regarded as vital detectors of brain inflammation and biomarkers of NP in mice, play a role in NP development by upregulating the expression of interleukins and kinases [16, 58]. In CCI rats, increased TRPV1 expression corresponds to the development of NP, while studies have shown that inhibiting TRPV1 upregulation can reduce NP levels in rats [21, 59–61]. We are the first to observe that both rTMS and TENS regulate TRPV1 expression within the central nervous system. Our investigations further revealed that both rTMS and TENS led to decreased TRPV1 levels in the PFC and spinal cord. Similar to the findings regarding inflammatory factors, TRPV1 levels in the rTMS group were significantly lower than those in the TENS group. This observation may partially explain the superior efficacy of rTMS over TENS in relieving NP.

In the present study, we examined the impact of rTMS and TENS on TRPV1 and inflammatory factors in the spinal cord. The development of mechanical nociception during CCI has further been suggested to involve sensitization linked to the upregulation of spinal TRPV1. While TENS primarily triggers analgesic effects through gate control mechanisms and endogenous opioid-like pathways, some studies have suggested a partial central analgesic mechanism for TENS [7, 62]. Our research aligns with these findings, demonstrating that peripheral TENS treatment can indeed regulate neuroinflammation in the spinal cord and PFC. In contrast, rTMS applied to the human DLPFC exerts a “top-down” inhibitory effect along the downstream pathway involving the midbrain–thalamus–cingulate gyrus, which is further mediated by descending fibers originating from the PFC to downregulate the expression of neuroinflammatory factors in the spinal cord [63]. Despite the distinct analgesic mechanisms involved in the central and peripheral neuromodulation strategies, both approaches can effectively mitigate neuroinflammation and alleviated mechanical nociception in the central nervous system. Notably, our analysis of the sciatic nerve tissue revealed that both rTMS and TENS improved sciatic nerve pathology and promoted sciatic nerve repair, suggesting that both central and peripheral stimulation can enhance sciatic nerve pathology. Overall, our study highlights the similar functions and potential mechanisms of rTMS and TENS, providing a theoretical basis for their combined use in mitigating NP.

## 5. Conclusion

In CCI rats, rTMS intervention in the PFC may be more effective at relieving NP than TENS applied to the peripheral nerves. Although rTMS may perform better than TENS, they may share similar potential mechanisms in the treatment of NP, which may be related to the downregulation of TRPV1 and control of neuroinflammation in the spinal cord and PFC.

## 6. Limitations

Our study has several limitations. First, we did not use a sham-operated group. As there was no sham operation group, the effects of CCI on neuroinflammation and TRPV1 expression could not be evaluated. However, according to our previous studies and those of other researchers [64, 65], the sham operation group did not exhibit any obvious neuroinflammation. We have confirmed in previous experiments that CCI causes neuroinflammation and increases TRPV1 expression, consistent with the findings of Ma et al. [66]. Our primary aim in this study was to compare the efficacy of TENS and rTMS in treating NP; therefore, we used only the CCI group as a negative control. Second, TRPV1 and inflammatory factors interact with each other. Regrettably, it is unknown whether TRPV1 affects the levels of inflammatory factors or whether inflammatory factors affect TRPV1 levels. In future studies, we plan to further verify the regulatory relationship between the two by blocking TRVP1 in the spinal cord and brain.

## Figures and Tables

**Table 1:** Primers used in this study.

Primer		Sequence
β-Actin	Forward	GAAGATCAAGATCATTGCTCC
	Reverse	TACTCCTGCTTGCTGATCCA

TRPV1	Forward	CTGCAAGCCACCTCCAGTCAAG
	Reverse	GACAGTGATGATAGGGCAGGAAGC

Abbreviation: TRPV1, transient receptor potential vanilloid type 1.

## Data Availability

Primary data material can be accessed by contacting the corresponding author.

## References

[1] Finnerup N. B., Kuner R., Jensen T. S. (2021). Neuropathic Pain: From Mechanisms to Treatment. *Physiological Reviews*.

[2] Zhang X., Guo H., Xie A., Liao O., Ju F., Zhou Y. K. (2020). MicroRNA-144 Relieves Chronic Constriction Injury-Induced Neuropathic Pain Via Targeting RASA1. *Biotechnology and Applied Biochemistry*.

[3] Bouhassira D. (2019). Neuropathic Pain: Definition, Assessment and Epidemiology. *Revue Neurologique*.

[4] Moisset X., Bouhassira D., Couturier J. A. (2020). Pharmacological and Non-Pharmacological Treatments for Neuropathic Pain: Systematic Review and French Recommendations. *Revue Neurologique*.

[5] Gilron I., Baron R., Jensen T. (2015). Neuropathic Pain: Principles of Diagnosis and Treatment. *Mayo Clinic Proceedings*.

[6] Moisset X., Bouhassira D., Attal N. (2021). French Guidelines for Neuropathic Pain: An Update and Commentary. *Revue Neurologique*.

[7] Mokhtari T., Ren Q., Li N., Wang F., Bi Y., Hu L. (2020). Transcutaneous Electrical Nerve Stimulation in Relieving Neuropathic Pain: Basic Mechanisms and Clinical Applications. *Current Pain and Headache Reports*.

[8] Attal N., Poindessous-Jazat F., De Chauvigny E. (2021). Repetitive Transcranial Magnetic Stimulation for Neuropathic Pain: A Randomized Multicentre Sham-Controlled Trial. *Brain: A Journal of Neurology*.

[9] Gopalsamy B., Farouk A. A. O., Tengku Mohamad T. A. S., Sulaiman M. R., Perimal E. K. (2017). Antiallodynic and Antihyperalgesic Activities of Zerumbone Via the Suppression of IL-1*β*, IL-6, and TNF-*α* in a Mouse Model of Neuropathic Pain. *Journal of Pain Research*.

[10] Liu X. J., Liu T., Chen G. (2016). TLR Signaling Adaptor Protein MyD88 in Primary Sensory Neurons Contributes to Persistent Inflammatory and Neuropathic Pain and Neuroinflammation. *Scientific Reports*.

[11] Toledo R., Stein D., Sanches P. (2021). RTMS Induces Analgesia and Modulates Neuroinflammation and Neuroplasticity in Neuropathic Pain Model Rats. *Brain Research*.

[12] Hu Y., Zhu Y., Wen X. (2022). Repetitive Transcranial Magnetic Stimulation Regulates Neuroinflammation, Relieves Hyperalgesia and Reverses Despair-Like Behaviour in Chronic Constriction Injury Rats. *European Journal of Neuroscience*.

[13] Lin H.-T., Chiu C.-C., Wang J.-J., Hung C.-H., Chen Y.-W. (2015). High Frequency Transcutaneous Electrical Nerve Stimulation With Diphenidol Administration Results in an Additive Antiallodynic Effect in Rats Following Chronic Constriction Injury. *Neuroscience Letters*.

[14] Kong W.-L., Peng Y.-Y., Peng B.-W. (2017). Modulation of Neuroinflammation: Role and Therapeutic Potential of TRPV1 in the Neuro-Immune Axis. *Brain, Behavior, and Immunity*.

[15] Feng Z., Pearce L. V., Zhang Y. (2016). Multi-Functional Diarylurea Small Molecule Inhibitors of TRPV1 With Therapeutic Potential for Neuroinflammation. *The AAPS Journal*.

[16] Malek N., Pajak A., Kolosowska N., Kucharczyk M., Starowicz K. (2015). The Importance of TRPV1-Sensitisation Factors for the Development of Neuropathic Pain. *Molecular and Cellular Neuroscience*.

[17] Ma W., Quirion R. (2007). Inflammatory Mediators Modulating the Transient Receptor Potential Vanilloid 1 Receptor: Therapeutic Targets to Treat Inflammatory and Neuropathic Pain. *Expert Opinion on Therapeutic Targets*.

[18] Saffarzadeh F., Eslamizade M. J., Ghadiri T., Modarres Mousavi S. M., Hadjighassem M., Gorji A. (2015). Effects of TRPV1 on the Hippocampal Synaptic Plasticity in the Epileptic Rat Brain. *Synapse*.

[19] Li R., Zhao C., Yao M., Song Y., Wu Y., Wen A. (2017). Analgesic Effect of Coumarins From Radix Angelicae Pubescentis Is Mediated by Inflammatory Factors and TRPV1 in a Spared Nerve Injury Model of Neuropathic Pain. *Journal of Ethnopharmacology*.

[20] Xue C., Liu S., Hu J. (2021). *Corydalis saxicola* Bunting Total Alkaloids Attenuate Paclitaxel-Induced Peripheral Neuropathy Through PKC*ε*/p38 MAPK/TRPV1 Signaling Pathway. *Chinese Medicine*.

[21] Wang J., Zhou F., Zhang S., Mao M., Feng S., Wang X. (2022). Participation of Transient Receptor Potential Vanilloid 1 in the Analgesic Effect of Duloxetine for Paclitaxel Induced Peripheral Neuropathic Pain. *Neuroscience Letters*.

[22] Huang Y. K., Lu Y. G., Zhao X. (2020). Cytokine Activin C Ameliorates Chronic Neuropathic Pain in Peripheral Nerve Injury Rodents by Modulating the TRPV1 Channel. *British Journal of Pharmacology*.

[23] Bennett G. J., Xie Y.-K. (1988). A Peripheral Mononeuropathy in Rat that Produces Disorders of Pain Sensation Like Those Seen in Man. *Pain*.

[24] Yang L., Wang S.-H., Hu Y., Sui Y.-F., Peng T., Guo T.-C. (2018). Effects of Repetitive Transcranial Magnetic Stimulation on Astrocytes Proliferation and nNOS Expression in Neuropathic Pain Rats. *Current Medical Science*.

[25] Huang J., Yang C., Zhao K. (2022). Transcutaneous Electrical Nerve Stimulation in Rodent Models of Neuropathic Pain: A Meta-Analysis. *Frontiers in Neuroscience*.

[26] Liu J., Zhuo H., Sun M. (2022). [Retracted] Rehabilitation of Post-Stroke Swallowing Dysfunction with Repeated Transcranial Magnetic Stimulation RTMS Based on Tomographic Images. *Contrast Media & Molecular Imaging*.

[27] Yu H., Liu S., Dai P., Wang Z., Liu C., Zhang H. (2022). Effects of Repetitive Transcranial Magnetic Stimulation on Gait and Postural Control Ability of Patients With Executive Dysfunction After Stroke. *Brain Sciences*.

[28] Krogh S., Aagaard P., Jønsson A. B., Figlewski K., Kasch H. (2022). Effects of Repetitive Transcranial Magnetic Stimulation on Recovery in Lower Limb Muscle Strength and Gait Function Following Spinal Cord Injury: A Randomized Controlled Trial. *Spinal Cord*.

[29] Lefaucheur J., Aleman A., Baeken C. (2020). Evidence-Based Guidelines on the Therapeutic Use of Repetitive Transcranial Magnetic Stimulation (rTMS): An Update (2014–2018). *Clinical Neurophysiology*.

[30] Zang Y., Zhang Y., Lai X. (2021). Repetitive Transcranial Magnetic Stimulation for Neuropathic Pain on the Non-Motor Cortex: An Evidence Mapping of Systematic Reviews. *Evidence-Based Complementary and Alternative Medicine*.

[31] Nardone R., Höller Y., Langthaler P. (2017). RTMS of the Prefrontal Cortex Has Analgesic Effects on Neuropathic Pain in Subjects With Spinal Cord Injury. *Spinal Cord*.

[32] Somers D. L., Clemente F. R. (2009). Contralateral High or a Combination of High- and Low-Frequency Transcutaneous Electrical Nerve Stimulation Reduces Mechanical Allodynia and Alters Dorsal Horn Neurotransmitter Content in Neuropathic Rats. *The Journal of Pain*.

[33] Cho H.-Y., Suh H. R., Han H. C. (2014). A Single Trial of Transcutaneous Electrical Nerve Stimulation Reduces Chronic Neuropathic Pain Following Median Nerve Injury in Rats. *The Tohoku Journal of Experimental Medicine*.

[34] Huang J., Gadotti V. M., Chen L. (2019). A Neuronal Circuit for Activating Descending Modulation of Neuropathic Pain. *Nature Neuroscience*.

[35] Ong W.-Y., Stohler C. S., Herr D. R. (2019). Role of the Prefrontal Cortex in Pain Processing. *Molecular Neurobiology*.

[36] Baliki M. N., Mansour A. R., Baria A. T., Apkarian A. V., Zang Y.-F. (2014). Functional Reorganization of the Default Mode Network Across Chronic Pain Conditions. *PLoS ONE*.

[37] Patrick Hardy S. G. (1985). Analgesia Elicited by Prefrontal Stimulation. *Brain Research*.

[38] Wang G. Q., Cen C., Li C. (2015). Deactivation of Excitatory Neurons in the Prelimbic Cortex Via Cdk5 Promotes Pain Sensation and Anxiety. *Nature Communications*.

[39] Martinez E., Lin H. H., Zhou H., Dale J., Liu K., Wang J. (2017). Corticostriatal Regulation of Acute Pain. *Frontiers in Cellular Neuroscience*.

[40] Lee M., Manders T. R., Eberle S. E. (2015). Activation of Corticostriatal Circuitry Relieves Chronic Neuropathic Pain. *The Journal of Neuroscience*.

[41] Liu Y., Xu H., Sun G. (2021). Frequency Dependent Electrical Stimulation of PFC and ACC for Acute Pain Treatment in Rats. *Frontiers in Pain Research*.

[42] Zhou H., Zhang Q., Martinez E. (2019). A Novel Neuromodulation Strategy to Enhance the Prefrontal Control to Treat Pain. *Molecular Pain*.

[43] Jiang X., Yan W., Wan R. (2022). Effects of Repetitive Transcranial Magnetic Stimulation on Neuropathic Pain: A Systematic Review and Meta-Analysis. *Neuroscience & Biobehavioral Reviews*.

[44] Shen Z., Li Z., Ke J. (2020). Effect of Non-Invasive Brain Stimulation on Neuropathic Pain Following Spinal Cord Injury: A Systematic Review and Meta-Analysis. *Medicine*.

[45] Li L., Huang H., Yu Y. (2022). Non-Invasive Brain Stimulation for Neuropathic Pain After Spinal Cord Injury: A Systematic Review and Network Meta-Analysis. *Frontiers in Neuroscience*.

[46] Mika J., Zychowska M., Popiolek-Barczyk K., Rojewska E., Przewlocka B. (2013). Importance of Glial Activation in Neuropathic Pain. *European Journal of Pharmacology*.

[47] Ellis A., Bennett D. L. H. (2013). Neuroinflammation and the Generation of Neuropathic Pain. *British Journal of Anaesthesia*.

[48] Fiore N. T., Austin P. J. (2019). Peripheral Nerve Injury Triggers Neuroinflammation in the Medial Prefrontal Cortex and Ventral Hippocampus in a Subgroup of Rats With Coincident Affective Behavioural Changes. *Neuroscience*.

[49] Yang Q., Li H., Zhang S. (2020). Red Nucleus IL-6 Mediates the Maintenance of Neuropathic Pain by Inducing the Productions of TNF-*α* and IL-1*β* Through the JAK2/STAT3 and ERK Signaling Pathways. *Neuropathology*.

[50] Rothman S. M., Huang Z., Lee K. E., Weisshaar C. L., Winkelstein B. A. (2009). Cytokine mRNA Expression in Painful Radiculopathy. *The Journal of Pain*.

[51] Hung A. L., Lim M., Doshi T. L. (2017). Targeting Cytokines for Treatment of Neuropathic Pain. *Scandinavian Journal of Pain*.

[52] Jiang R., Li P., Yao Y. X. (2019). Pulsed Radiofrequency to the Dorsal Root Ganglion or the Sciatic Nerve Reduces Neuropathic Pain Behavior, Decreases Peripheral pro-Inflammatory Cytokines and Spinal *β*-Catenin in Chronic Constriction Injury Rats. *Reg Anesth Pain Med*.

[53] Wang X., Tian S., Wang H. (2020). Botulinum Toxin Type A Alleviates Neuropathic Pain and Suppresses Inflammatory Cytokines Release From Microglia by Targeting TLR2/MyD88 and SNAP23. *Cell & Bioscience*.

[54] Matsuo H., Uchida K., Nakajima H. (2014). Early Transcutaneous Electrical Nerve Stimulation Reduces Hyperalgesia and Decreases Activation of Spinal Glial Cells in Mice With Neuropathic Pain. *Pain*.

[55] Caterina M. J., Leffler A., Malmberg A. B. (2000). Impaired Nociception and Pain Sensation in Mice Lacking the Capsaicin Receptor. *Science*.

[56] Frias B., Merighi A. (2016). Capsaicin, Nociception and Pain. *Molecules*.

[57] Palazzo E., Luongo L., de Novellis V., Berrino L., Rossi F., Maione S. (2010). Moving Towards Supraspinal TRPV1 Receptors for Chronic Pain Relief. *Molecular Pain*.

[58] Marrone M., Morabito A., Giustizieri M. (2017). TRPV1 Channels Are Critical Brain Inflammation Detectors and Neuropathic Pain Biomarkers in Mice. *Nature Communications*.

[59] Guo S., Lin J., Huang L. (2019). Silencing of Spinal Trpv1 Attenuates Neuropathic Pain in Rats by Inhibiting CAMKII Expression and ERK2 Phosphorylation. *Scientific Reports*.

[60] Wang Z., Ling D., Wu C., Han J., Zhao Y. (2020). Baicalin Prevents the Up-Regulation of TRPV1 in Dorsal Root Ganglion and Attenuates Chronic Neuropathic Pain. *Veterinary Medicine and Science*.

[61] Xing F., Gu H., Niu Q. (2019). MZF1 in the Dorsal Root Ganglia Contributes to the Development and Maintenance of Neuropathic Pain Via Regulation of TRPV1. *Neural Plasticity*.

[62] Lin T., Gargya A., Singh H., Sivanesan E., Gulati A. (2020). Mechanism of Peripheral Nerve Stimulation in Chronic Pain. *Pain Medicine*.

[63] Leung A., Metzger-Smith V., He Y. (2018). Left Dorsolateral Prefrontal Cortex rTMS in Alleviating MTBI Related Headaches and Depressive Symptoms. *Neuromodulation: Technology at the Neural Interface*.

[64] Zhu J., Yang J., Xu J. (2021). MiR-223 Inhibits the Polarization and Recruitment of Macrophages Via NLRP3/IL-1*β* Pathway to Meliorate Neuropathic Pain. *Pain Research and Management*.

[65] Zhang D., Mou J-Ying, Wang F., Liu J., Hu X. (2019). CRNDE Enhances Neuropathic Pain Via Modulating miR-136/IL6R Axis in CCI Rat Models. *Journal of Cellular Physiology*.

[66] Ma Y., Deng Q., Li S. (2021). Targeted by miR-338-3p, Induces Neuropathic Pain by Interacting With NECAB2. *Journal of Molecular Neuroscience*.

